# Editorial: Optimal Mobility and Function Across the Lifespan

**DOI:** 10.3389/fphys.2021.652640

**Published:** 2021-02-09

**Authors:** David A. Hart, Ronald F. Zernicke

**Affiliations:** ^1^Faculty of Kinesiology, University of Calgary, Calgary, AB, Canada; ^2^McCaig Institute for Bone & Joint Health, University of Calgary, Calgary, AB, Canada; ^3^Department of Surgery, University of Calgary, Calgary, AB, Canada; ^4^Alberta Health Services, Bone & Joint Health Strategic Clinical Network, Edmonton, AB, Canada; ^5^Department of Orthopedic Surgery, Michigan Medicine, University of Michigan, Ann Arbor, MI, United States; ^6^School of Kinesiology, University of Michigan, Ann Arbor, MI, United States; ^7^Department of Biomedical Engineering, University of Michigan, Ann Arbor, MI, United States; ^8^Exercise & Sport Science Initiative, University of Michigan, Ann Arbor, MI, United States; ^9^Department of Physiology and Pharmacology, University of Calgary, Calgary, AB, Canada

**Keywords:** mobility, cardiovascular, exercise physiology, musculoskeletal, aging

Mobility and the ability to functionally navigate the environment are intrinsically ingrained in the evolution leading to *Homo sapiens*. Likewise, mobility and a threshold level of exercise are essential across the lifespan from birth to old age to optimize growth and maturation early, and then to avoid deconditioning with advancing age. Connective tissues including bones, muscles, tendons, ligaments, cartilage, and menisci require mobility to maintain optimal integrity (i.e., “use it or lose it” paradigm) and need to work within the boundary conditions of Earth. In addition, it is not only the human musculoskeletal (MSK) system that depends on mobility and exercise to maintain its integrity, but also the cardiovascular system (CVS), and brain and other highly vascularized systems (e.g., kidneys and lungs). The articles in this Research Topic focus on understanding the role and effects of mobility and exercise in maintaining multiple physiological systems and avoiding the deconditioning that can accompany advancing age. The articles can be grouped into Three Themes: (1) Overview of how exercise is ingrained in human physiology, (2) Examination of how exercise can be assessed and used as an approach for disease prevention and risk mitigation in younger individuals, and (3) Examples of the effectiveness of exercise for enhancing successful aging. Given the “graying” of many global populations, the development of chronic diseases in the elderly, and the need to both prevent loss of mobility (i.e., Exercise is Health), as well as using mobility and exercise to mitigate the impact of early chronic diseases (i.e., Exercise is Medicine), many of the Research Topic articles focus on the elderly.

## Theme One

Hart and Zernicke provide an overview of how mobility and exercise (patterned mobility) were ingrained into multiple physiologic systems during the evolutionary process leading to *Homo sapiens*. Mobility and exercise are critical for growth and maturation through to skeletal maturity as humans develop the ability to function in Earth's environment with its attendant gravitational forces. How biomechanics and biology are integrated to yield long-term functioning is also addressed.

## Theme Two

The systematic review by Patel et al. focuses on non-elite sport involvement and bone density in younger individuals (11–35 years). Peak bone density is acquired early in life, and enhanced bone density can reduce risk of osteoporotic fractures later in life, particularly for post-menopausal females, who comprise~75% of the elderly with osteoporosis (OP). The analysis by Patel and colleagues showed that weight-bearing sport involvement has a beneficial effect on calcaneal bone density, which may also portend beneficial effects on other skeletal tissues.

Tuttor et al. compared two different exercise protocols designed to impact cardiometabolic risk factors in overweight “middle aged” males. Both protocols were effective, but a high-intensity resistance exercise was more effective. Two key findings emerged: (1) different exercise protocols can effectively have a positive influence on the CVS, and (2) variations exist between individuals regarding which type of exercise will yield the optimal response. As Hart and Zernicke note, it is known that some people respond better to resistance than aerobic exercise and vice versa.

Assessing how individuals walk in different environments (i.e., controlled vs. uncontrolled) can provide insights into how mobility is influenced by navigation cues, and how this may be also influenced by age. Renggli et al. indicate that controlled laboratory assessments yield different results when compared to uncontrolled mobility (i.e., gait) in real-world environments assessed using wearable measurement devices. For assessing both young and elderly individuals, it is important to understand the type and intensity of mobility activity, as well as the environment in which the activity occurs. An uncontrolled environment likely requires incorporation of navigation cues more so than a laboratory setting.

The final article in Theme Two bridges Themes Two and Three. Mancinelli et al. examine the ability of neuromuscular electrical stimulation (NMES) to modify the muscles of the healthy elderly, using the *vastus lateralis* muscle of active, healthy older individuals. As older individuals may develop sarcopenia, which can impede mobility, using NMES to enhance muscle integrity could lead to more effective mobility and, thus, exert greater impact on physiological systems at risk due to a lack of mobility. Given the effectiveness of the intervention, one could also postulate that application of NMES to younger individuals may prevent or inhibit the development of some forms of sarcopenia.

## Theme Three

Theme Three articles focus on the elderly and address issues in the context of mobility and successful, healthy aging.

The first contribution focuses on the MSK system in the elderly and how to optimize its integrity. Endo et al. proposed to optimize skeletal muscle fitness using resistance training in the elderly. As noted earlier, during the aging process many individuals develop sarcopenia leading to impaired mobility, and Endo et al. used exercise—alone or in combination with other modalities—in an effort to blunt the potential for developing sarcopenia. Tavoian et al. also focus on resistance exercise in the elderly to diminish the risk for development of chronic diseases, and their results support the premise that resistance exercise is more beneficial than aerobic exercise. Hill et al. compare the advantages of downhill walking vs. level walking for the retention of muscular and physical function in the elderly. In aggregate, these three studies indicate that it is not just exercise that is important, but the type of exercise can also be important. The next article focuses on using SPARC metrics to assess variations in the “timed up-and-go” test in individuals over 80 years, who were at risk for falls vs. those who were not (Figueiredo et al.). As falls are a leading cause of low impact hip fractures, being able to predict who may be at risk with a relatively simple test could lead to preventative measures to mitigate such risk. Finally, building on the theme of mitigating risk for adverse events in the elderly, including falls, Freiberger et al. provide a narrative review focused on community-dwelling elderly and the risks presented by age-related loss of cognition, physical deconditioning, and other mobility limitations.

A cluster of articles in Theme Three focuses on the role of mobility and exercise in the elderly related to bone and CVS health. Shojaa et al. provide a systematic review and meta-analysis of exercise on bone health in post-menopausal females. They evaluate and summarize existing research on optimal exercise protocols to use and their outcomes in this population. As osteoporosis (OP) is a prevalent disease in this cohort of women (~75% of all OP), and OP can predispose individuals to hip fractures and other fractures that can lead to morbidity and mortality, optimizing preventative measures is significant. With respect to the interactions among CVS health, exercise, and successful aging, a subset of three review articles is included in Theme Three. Santos and Umpierre address the links between exercise and risk for atherosclerosis and as such emphasize not only the elderly, but also those in younger age groups. The advantages and impact of exercise, as well as current gaps in information that need additional study, indicate that while some concrete evidence exists in this area, much research remains to be done. de Oliverira Sant'Ana et al. generated a review focused on the role of exercise as a preventative measure in the healthy elderly. As such, the exercise protocols were designed to maintain the integrity of the CVS and minimize the risk for deconditioning and emergence of disease (i.e., Exercise is Health). Finally, Xing et al. provide a mini-review that focuses on the role of exercise and mobility on recovery from a CVS event (i.e., myocardial infarction) (i.e., Exercise is Medicine). Collectively, extant data suggest that exercise protocols can have a positive impact on both prevention, as well as recovery from a loss of health as it pertains to the CVS.

### Integrating Ideas

The collection of articles in this Research Topic support the concept that humans require mobility to maintain the integrity of the musculoskeletal system (MSK), as well as the CVS. Because CVS integrity is central to many physiological systems (e.g., brain, kidneys, and lungs), there is a “cascade effect” for optimal health that can be traced back to the need to optimally maintain the MSK system and retain its functionality across the lifespan (Hart and Zernicke). In sum, Prevention is better than Treatment for minimizing risk for loss of function for many human physiological systems.

## Author Contributions

DH wrote the initial draft of this Editorial. DH and RZ edited and polished the article. Both authors agree with the final version and its submission to Frontiers.

## Conflict of Interest

The authors declare that the research was conducted in the absence of any commercial or financial relationships that could be construed as a potential conflict of interest.

